# Measuring gravity with milligram levitated masses

**DOI:** 10.1126/sciadv.adk2949

**Published:** 2024-02-23

**Authors:** Tim M. Fuchs, Dennis G. Uitenbroek, Jaimy Plugge, Noud van Halteren, Jean-Paul van Soest, Andrea Vinante, Hendrik Ulbricht, Tjerk H. Oosterkamp

**Affiliations:** ^1^ Leiden Institute of Physics, Leiden University, P.O. Box 9504, 2300 RA Leiden, Netherlands.; ^2^ Istituto di Fotonica e Nanotecnologie, CNR and Fondazione Bruno Kessler, I-38123 Povo, Trento, Italy.; ^3^ School of Physics and Astronomy, University of Southampton, SO17 1BJ Southampton, UK.

## Abstract

Gravity differs from all other known fundamental forces because it is best described as a curvature of space-time. For that reason, it remains resistant to unifications with quantum theory. Gravitational interaction is fundamentally weak and becomes prominent only at macroscopic scales. This means, we do not know what happens to gravity in the microscopic regime where quantum effects dominate and whether quantum coherent effects of gravity become apparent. Levitated mechanical systems of mesoscopic size offer a probe of gravity, while still allowing quantum control over their motional state. This regime opens the possibility of table-top testing of quantum superposition and entanglement in gravitating systems. Here, we show gravitational coupling between a levitated submillimeter-scale magnetic particle inside a type I superconducting trap and kilogram source masses, placed approximately half a meter away. Our results extend gravity measurements to low gravitational forces of attonewton and underline the importance of levitated mechanical sensors.

## Teaser

A levitated submillimeter scale magnetic particle inside a type I superconducting trap detects gravitational coupling.

## INTRODUCTION

Einstein’s theory of general relativity (GR), our widely accepted theory of gravity, has seen different experimental confirmations (*1*, *2*) by observing massive astronomical objects and their dynamics, most recently by the direct observation of gravitational waves from the merger of two black holes (*3*) and the imaging of a black hole by the event horizon telescope (*4*), as well as dedicated satellite missions for testing the basic principle of GR—the equivalence principle (*5*) and frame dragging effects (*6*). Laboratory experiments have been continuously increasing the sensitivity of gravity phenomena, including general relativistic effects in atom clocks and atom interferometers (*7*, *8*), tests of the equivalence principle (*9*, *10*), precision measurements of Newton’s constant (*11*, *12*), and tests of the validity of Newton’s law at micrometer-scale distances (*13*, *14*).

However, gravity has never been tested for small masses and on the level of the Planck mass. Measurements of gravity from classical sources in laboratory table-top settings is contrasted by an increasing interest to study gravitational phenomena originating from quantum states of source masses, for example, in the form of the gravitational field generated by a quantum superposition state (*15*–*19*). The effort ultimately aims at directly probing the interplay between quantum mechanics and GR in table-top experiments. Because quantum coherence is easily lost for increasing system size, it is important to isolate gravity as a coupling force for as small objects as possible, which in turn means to measure gravitational forces and interactions extremely precisely.

At the same time, massive quantum sensors are especially suited for tests in a regime with appreciable gravitational influences, which is favorable in probing fundamental decoherence mechanisms related to gravity (*20*, *21*) or proposed physical models of the wave function collapse (*22*–*24*) featuring the system mass explicitly, such as the continuous spontaneous localization model (*25*) and the Diósi-Penrose model of gravitationally induced collapse (*26*–*28*).

An emerging technology for ultrasensitive sensing is based on levitated mechanical systems. These can be used for the mechanical sensing of very weak forces and to probe quantum physics at increasing scales of mass (and space). In optical levitation schemes, the heating from trapping lasers is the most prominent source of noise. Worse, in any quantum experiment, they will provide a source of decoherence, greatly increasing the difficulty of creating macroscopic quantum states. In magnetically levitated systems, this pathway of decoherence is largely removed (*29*).

The extremely low damping of magnetic systems, combined with their relatively high mass and operation in low-noise cryogenic environments, makes them well suited for mesoscopic probes of quantum mechanics and could provide a test to possible limits of the applicability of quantum mechanics to the macroscopic world (*30*, *31*). Van Waarde *et al.* (*32*) and Vinante *et al.* (*33*) have previously realized such magnetic levitation of submilligram particles, in which the motional state of the particle is read out by means of superconducting quantum interference device (SQUID) detection.

In a recent publication, Westphal *et al.* (*34*) have demonstrated gravitational coupling between two 90-mg, 1-mm-radius, gold spheres, achieved off resonance at millihertz frequencies in a torsion balance-type geometry. Recent work by Brack *et al.* (*35*) has shown the dynamical detection of gravitational coupling between two parallel beams of a meter in size in the hertz regime. Here, we present work with a 2.4-kg source mass and a magnetically levitated submilligram test mass, giving a coupling of 1030 aN with a force noise of $0.5\;\text{fN}/\sqrt{\text{Hz}}$ . This work provides an intermediate step toward an experiment where a small test mass senses the gravity sourced by a small source mass.

## RESULTS

**Fig. 1.**
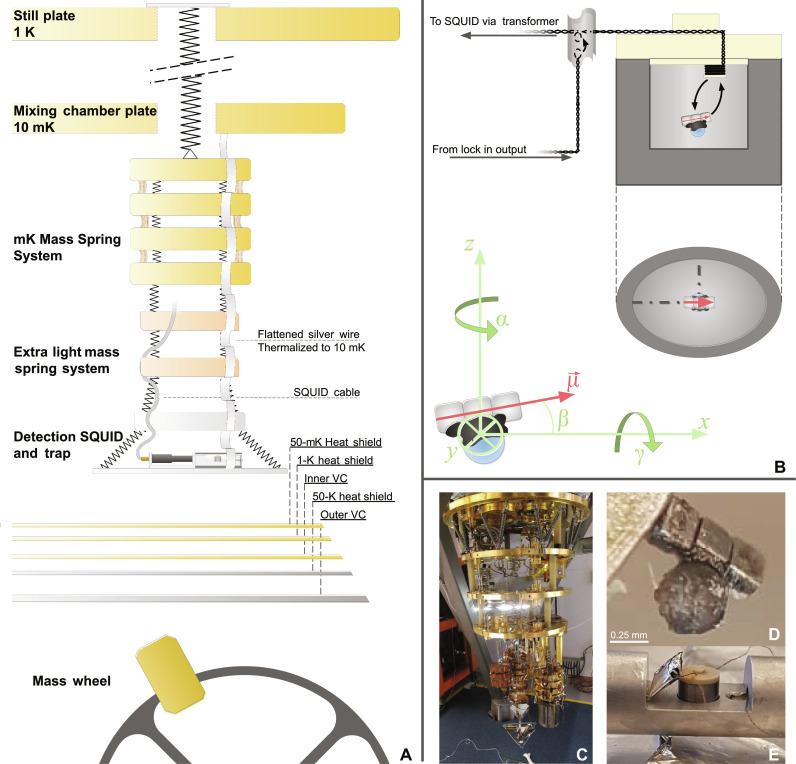
Schematic depiction of the experimental setup. (**A**) Multistage mass spring system to isolate from external vibrations, as discussed in the text. Electromagnetic shielding of the trap is discussed in the Supplementary Materials A. (**B**) Conventions for degrees of freedom adopted from Vinante *et al.* (*33*). Detection by SQUID as discussed in the text. Calibration loop as discussed in the text and Supplementary Materials C. (**C**) An image of the dilution refrigerator used for the experiments, including the multistage mass spring system. (**D**) The magnetic particle, composed of three 0.25 mm by 0.25 mm by 0.25 mm Nd_2_Fe_14_B magnets (SuperMagnetMan, C0005-10) magnetically attached end-to-end and a single spherical glass bead with a 0.25-mm radius attached using Stycast to the middle of the magnets, which is used to break the symmetry of the γ mode. (**E**) The trap, as placed in the aluminum holder without the shielding cylinder. The aluminum foil envelope provides additional electromagnetic shielding between the calibration transformer and the pick-up loop. Further details and images of the setup are shown in Supplementary Materials A.

The core of the setup is a type I superconducting trap with a magnetic particle levitated therein, as shown in Fig. 1B. The trap is made of tantalum with a critical temperature of *T*_c_ = 4.48 K. We perform the experiment at temperatures below 100 mK. The trap has an elliptical shape (4.5 mm by 3.5 mm, with the height from bottom of the trap to the coil being 4.7 mm) to confine the modes of the levitated magnetic particle to the axial system of the trap. The particle is composed of a set of three 0.25 mm by 0.25 mm by 0.25 mm Nd _2_Fe_14_B magnets that are magnetically attached north-to-south as also shown in Fig. 1B, and a spherical glass bead, 0.25-mm radius, that is attached to the middle magnet using stycast. This bead is added to break the rotational symmetry of the zeppelin around the *x* axis (angle γ). Typical remnant magnetisation of Nd _2_Fe_14_B magnets is in the order of 1.4 T. The estimated mass of the full particle, as depicted in Fig. 1D, is 0.43 mg. Using the infinite plane approximation of Vinante *et al.* (*33*), we calculate an expected z-mode frequency of 27 Hz in this geometry.

The motion of the particle results in a change of flux through a loop at the top of the trap (the pick-up loop), which is detected using a two-stage biased SQUID coupled inductively to the pick-up loop. The loop is positioned off-center so that the symmetry is broken, and all modes couple to the loop. A third loop positioned halfway between the SQUID input loop and the pick-up loop is coupled inductively to a calibration loop. This transformer is used to calibrate the energy coupling β^2^ between the detection circuit and the degrees of motion of the zeppelin, providing calibrated motion of the zeppelin from the measured flux signal. This procedure is further described in Supplementary Materials C.

We suspend the setup from springs in a multistage mass spring system to shield the experiment from external vibrations, both vertical and lateral. The bottom three masses (one aluminum, two copper) are similar in weight to the experimental setup, with a lowest resonance frequency at 0.9 Hz. Above that is a millikelvin mass spring system with a lowest resonance frequency of 4.8 Hz. We refer to (*36*) for more details on a near identical mass-spring system and its performance in a similar dilution refrigerator. This combination is suspended from the 1 K plate by a long spring. Thermalization of the experiment is provided by a flattened silver wire, which is mechanically soft while providing a good thermal link. This entire system is depicted in Fig. 1A.

The cryostat as a whole is rigidly attached to a 25–metric ton concrete block, which is again placed on pneumatic dampers to limit vibrations coupling in from the building. The pulse tube cooler and the vacuum pumps for the circulation of the mixture are rigidly attached to the building through a second frame and attached to the cryostat only by edge welded bellows and soft copper braiding to further limit external excitations from reaching the particle.

To demonstrate the force sensitivity of the system and as a proof of concept for gravitational coupling in levitated magnetic systems, we used an electrically driven wheel with a set of three 2.45-kg brass masses, placed equally spaced along the outer rim. This wheel was used to create a time-dependent gravitational gradient at the resonant frequency of a selected mode of the zeppelin, in an effort to drive the motion gravitationally. The frequency of the masses was read out optically using a laser and photodiode, in which the masses act as a mechanical shutter.

**Fig. 2.**
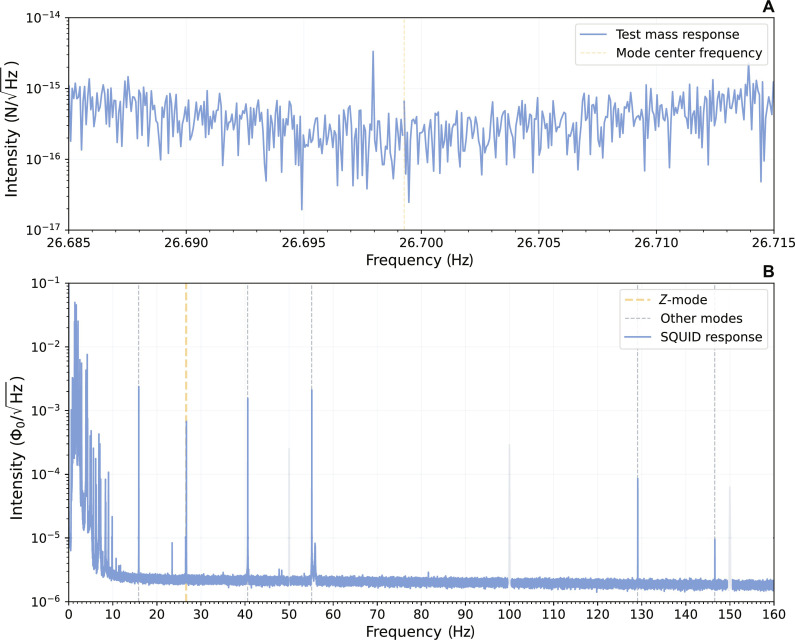
Levitated particle spectra. (**A**) Force noise of the 27-Hz mode when gravitationally driven at 1.3-mHz detuning, overnight. (**B**) Typical resonator power spectrum. In this figure, we have grayed out the regular 50-Hz European electrical noise. This noise typically has a similar power to the particle resonances.

In Fig. 2B, we show the uncalibrated spectrum. We clearly observe the six different modes corresponding to the three translational modes and three rotational modes respectively. Furthermore, we see a distinct peak at 27 Hz, which we attribute to the z-mode as discussed in the beginning of this section. These modes were validated and calibrated by performing a magnetic drive, excited by a flux injected through the calibration transformer, as shown in Fig. 1B.

Using this magnetic drive, we determine the decay time of the modes during the subsequent ringdown. For the 26.7-Hz mode, we find a lower bound τ = 1.09 × 10^5^ s, or a *Q* factor of *Q* = 9.13 × 10^6^. This procedure is further discussed in Supplementary Materials B. For the other modes, we arrive at *Q* factors that are about an order of magnitude lower.

We test the force sensitivity of our mode by driving the 26.7-Hz mode using the brass masses of the mass wheel. The resulting excitation at one position of the wheel in the force spectrum, is shown in Fig. 2A. The calibration of this spectrum is discussed in the Supplementary Materials. The resulting force noise of this z-mode is approximately $0.5\;\text{fN}/\sqrt{\text{Hz}}$ , or equivalently, a displacement noise of $60\;\text{pm}/\sqrt{\text{Hz}}$ , in an 8-mHz bandwidth centered around the orange dotted line that indicates the frequency of the resonance. Equivalently, we can determine the motion of the trap in which the particle is levitated by dividing the force noise by the spring constant of the confinement potential that keeps the particle around its equilibrium height. The spring constant for the z-mode was determined to be *k* = 12 × 10^−3^ N/m, resulting in a trap displacement noise of $30\;\text{fm}/\sqrt{\text{Hz}}$ . This vibrational noise is not yet thermally limited but rather corresponds to a mode temperature of 3 K, which we attribute to the limits of the vibration isolation inside the cryostat.

**Fig. 3.**
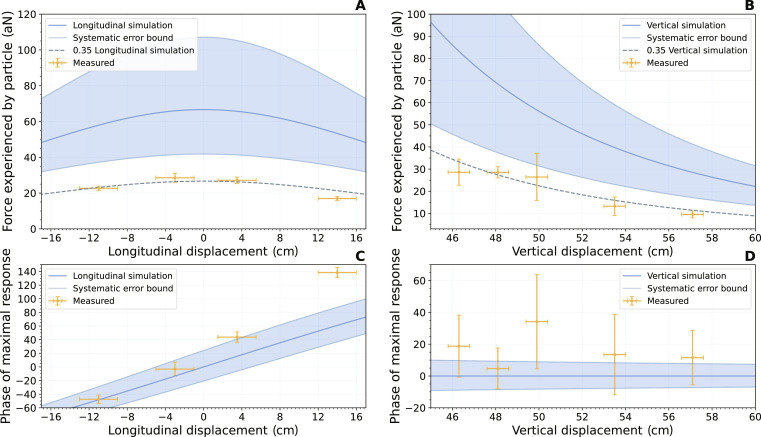
Response to gravitational drive as function of separation. (**A**) Force experienced by the mechanical resonator as a result from drive using the mass wheel, for different lateral displacement of the mass wheel relative to the particle. The dashed blue line represents the simulated gravitational force at the position of the magnetic particle as discussed in the main text. The blue area denotes the systematic uncertainty discussed in the text. A second, dashed line is plotted, which has a scaling factor of 0.35 applied, that seems to agree with the data more closely, as discussed in the main text. (**B**) Similar to (A), but now for a vertical displacement of the mass wheel relative to the particle, when the wheel is kept centered below the particle. Systematic bounds as discussed in the text. (**C**) Here, we see the wheel phase at which the magnetic particle experiences the strongest force, plotted against 00 longitudinal displacement. (**D**) Similar to (C), but now for a vertical displacement.

In Fig. 3, we show the measured gravitational interaction for different displacements of the wheel, using the method described in Supplementary Material E. We also plot the phase of the masses along the wheel rotation for which the particle experiences maximal force; see fig. S2. For the longitudinal displacement, the vertical displacement was held at 48 ± 4 cm. In the run of vertical positions, the wheel was kept centered with respect to the trap. Included is the expected gravitational signal at the location of the magnetic particle for the z-mode of the particle, which was calculated from an analytical simulation where the mass was taken to consist of multiple point masses. From this same simulation, a systematic error bound was derived, on the basis of an estimated systematic error, for the longitudinal run, of ±5 cm longitudinal, ±3 cm lateral, and ±4 cm vertical, which were estimated from the geometry of the wheel, the mass spring system and the systems used to measure the displacements. For the vertical run, the bounds are ±2 cm in each principal direction, based on the increased stability of the system under vertical displacement. The observed signal agrees with the simulated force signal to within a factor 0.35 with an SE of 0.02, which was determined by means of an orthogonal distance regression fit to the data.

## DISCUSSION

We attribute this constant factor to the effect of the wheel on the motion of the trap and its holder. The trap and its platform also experience a gravitational pull. For the sake of its vibration isolation, the trap and its platform are suspended by springs, and thus, the platform is set in motion by the gravitational pull on it. The amplitude of this motion is extra small, however, because the frequency of the gravitational drive is approximately a factor of 10 above the resonance of the suspension. This naturally gives rise to a 180° phase shift in the response of the trap’s motion. The small motion of the trap cause the walls of the trap to exert a small force on the particle. Because of the 180° phase shift, this force is out of phase with the gravitational pull that the particle experiences. Thus this leads to a suppression of the particle response. The suppression depends on the total force on the platform, the gravitational gradient due to the wheel, the angle which the platform makes with respect to the horizontal, etc. Additional complicating factors are the changing stiffness of the SQUID cable when cooling down, which can lead to a substantial tilt of the platform.

We demonstrate the detection of a 30-aN gravitational signal at 27-Hz and a damping linewidth as low as γ/2π = 2.9 μHz, with a 0.43-mg test mass, paving the way for future experiments in which both source and test mass are in this regime. This work could be used to derive a more stringent bound on dissipative collapse models. Furthermore it provides a promising platform to test for possible deviations from inverse-square force laws and fifth-force models (*37*, *38*), theories of modified Newtonian dynamics (*39*, *40*), and other extensions of the standard model (*41*).

By ensuring that the pick-up loop is placed off-center with respect to the trap and by breaking the rotational symmetry, we demonstrate detection of all six mechanical modes, in comparison to earlier work. As we will discuss in future work, this is critical to the stability of the mode under test due to nonlinear mixing between the different modes.

With a mode temperature of 3 K compared to an operating temperature of 30 mK, we are currently not yet thermally limited. It would require another 20 dB of vibration isolation to reach thermal motion.

By using a second particle in a different trap as source mass, or a similar construction, this work paves the way toward easily scalable measurements of gravitational coupling in the hertz regime and with source masses at Planck mass level, ultimately allowing for testing gravity in a yet unexplored low-mass regime and pushing into the quantum controlled domain. Coupling of the detection SQUIDs in this scheme to an superconducting inductor capacitor (LC) circuit would provide a means of inserting single microwave photons, providing access to the toolbox of quantum-state manipulation. This would further extend this work toward truly macroscopic superposition measurements and possibly gravitationally induced entanglement.
