# Supplementary material for: Measuring gravity with milligram levitated masses
Source: Sci Adv. 2024 Feb 23;10(8):eadk2949. doi: 10.1126/sciadv.adk2949 (PMC10889343; doi:10.1126/sciadv.adk2949)
Supplement: Supplementary file 1 — Supplementary Text Figs. S1 to S8 Table S1 [file sciadv.adk2949_sm.pdf]

Science Advances
AAAS

Supplementary Materials for

Measuring gravity with milligram levitated masses

Tim M. Fuchs *et al.*

Corresponding author: Tjerk H. Oosterkamp, oosterkamp@physics.leidenuniv.nl

Sci. Adv. **10**, eadk2949 (2024)
DOI: 10.1126/sciadv.adk2949

This PDF file includes:

Supplementary Text
Figs. S1 to S8
Table S1

Supplementary Materials A Further Schematics and Photographs of the Setup

Since the gravitational coupling is small, great care was taken to shield the experiment from magnetic and electric forces. The heat- and vacuum shields of the cryostat are made of several millimeters of gold plated copper, providing a high level of shielding with respect to electrical forces. Several layers of aluminium foil were wound around the heat shields that achieve temperatures below the critical temperature of aluminium (namely, the 1 K shield, and the 50 mK shield). This was done in an effort to provide some basic shielding from stray magnetic forces. Further magnetic shielding was incorporated in the holder of the experiment, as shown in figure S1.

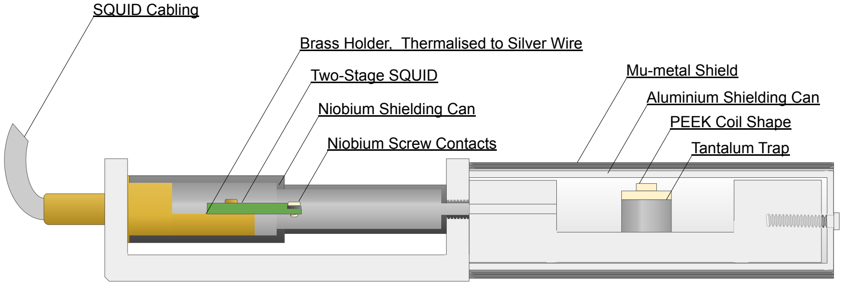

Fig. S1: **Close-up of the experiment holder in Fig. 1**. The SQUID detection chip is housed in a niobium can (respectively, Magnicon CAR-1 Two-Stage SQUID and NC-1 Can) that provides shielding from AC magnetic fields through the Meissner effect. The niobium can is screwed into the larger aluminium holder, which similarly provides AC magnetic shielding to the trap through the Meissner effect. The tantalum trap is capped with a PEEK coil-shape, placed offset from the center of the trap, around which the pick-up loop is wound. Additional shielding from DC magnetic fields is provided by several layers of mu-metal foil wrapped around the aluminium holder. This shielding was added under the assumption that stray magnetic fields influence the position of the magnetic particle within the trap and otherwise would get 'frozen-in' to the superconductors as they cool down.

In figure S2 we show a schematic to further elucidate the axial system used in the gravitational measurements, showing the longitudinal direction, the vertical direction and the wheel phase over which the wheel was displaced.

The factor n follows from the detection by the laser-photodiode combination not distinguishing between masses.

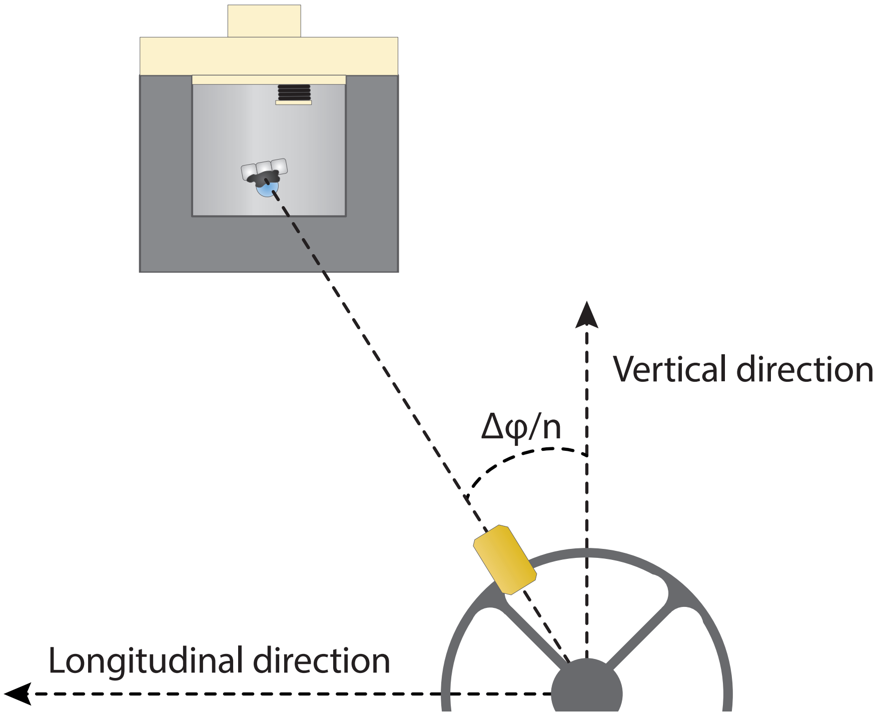

Fig. S2: **Schematic depicting the phase angle of the wheel**, as used to define the phase of maximal response in figure 3. Here, n refers to the amount of masses on the wheel, in this case three. This factor follows from the detection not distinguishing between the different masses.

In figure S3 are some photographs of critical elements of the experiment. Namely, (part of) the vibration isolation, the coil used to detect the motion of the particle, the transformer used to calibrate the coupling, the tantalum trap, and the wheel used to source the gravitational signal.

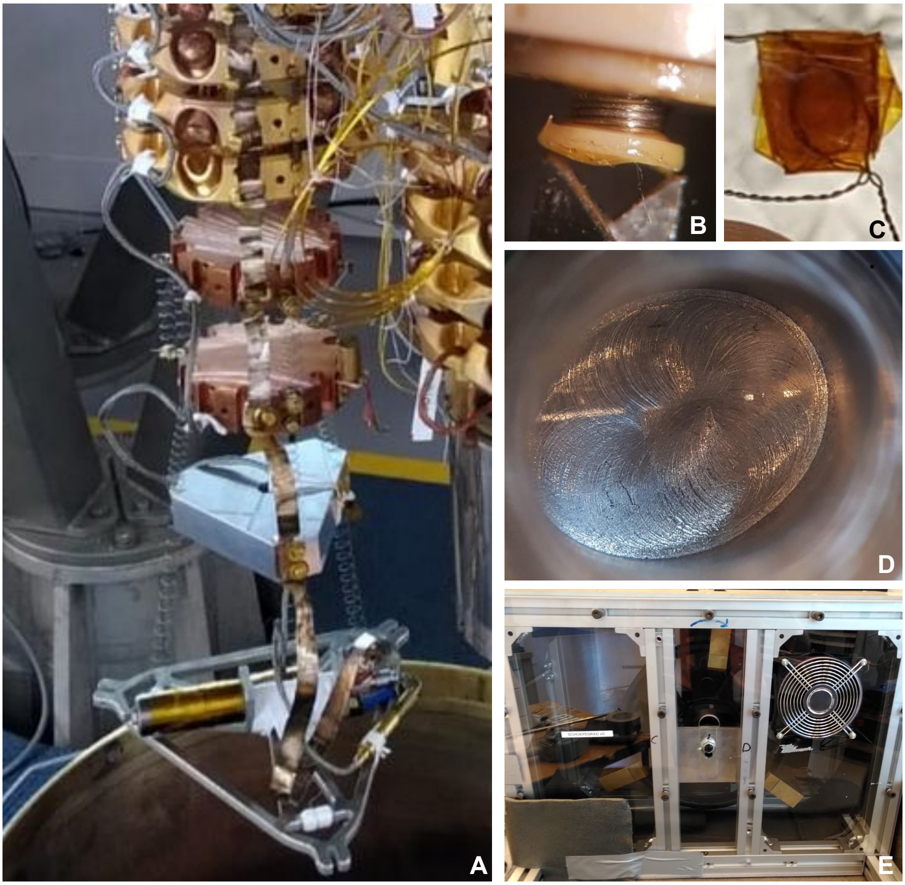

Fig. S3: **Photos of the the experimental apparatus of Fig.1**.
A: The mass-spring system and the silver wire used for thermalisation of the masses and the experiment, with at the bottom the holder on a small triangular platform to attach the springs. ***B***: The coil after the first two layers of five loops were wound around it, with the rest of the cap above. The final pick-up loop consisted of four layers. ***C***: A close-up of the calibration transformer, before aluminium shielding was added around it. ***D***: The tantalum trap, with the elliptical shape and milling marks visible. ***E***: The 'mass-wheel' as used in the experiments to show gravitational coupling. The three brass masses are placed in an equilateral triangle. Not shown is the laser-photodiode combination used to read out the frequency of the masses. The wheel is surrounded with one centimeter thick steel plates and is attached to a bridge made out of MK-profiles, which is used to control the elevation and positioning of the wheel in the lab relative to the trap inside the cryostat.

Supplementary Materials B Determination of decay time, damping factor, and the quality factor of the mechanical modes

The exponential decay time (τ) of each mode was determined through ringdown measurements at high amplitude. At low vibrational amplitude, the measured τ increases, as is expected from non-linearities in the trapping potential. High amplitude measurements were taken to provide a lower bound on τ with a high signal-to-noise ratio. We focus on the 27 Hz mode, as this is the mode we used to detect our gravitational signal. As discussed in section 2, this mode corresponds most closely to the theoretically expected z-mode frequency. Furthermore, it showed the highest degree of vibrational isolation, which is also as expected given our implemented vibration isolation functions predominantly in z. From the response in phase and amplitude shown in section 2 this is further verified, matching closely to the expected scaling of the z-mode gravitational coupling (both amplitude and phase) under translation of the gravitational source.

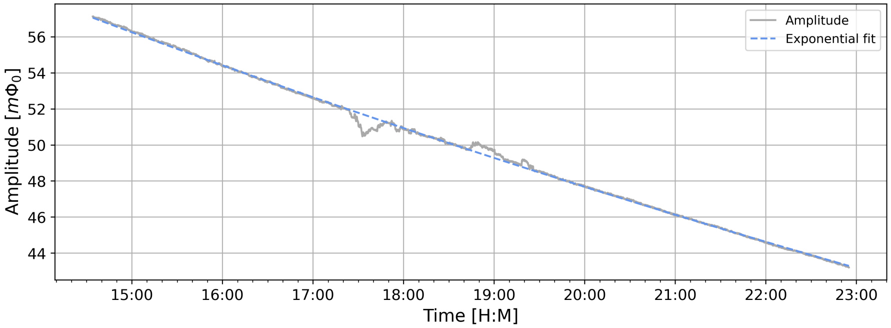

Fig. S4: **A typical ringdown of the 27 Hz mode**, as performed after a magnetic drive through the calibration transformer. The decay time (τ) is extracted from an exponential fit.

The high amplitude τ provides a lower bound to the low amplitude τ of the system. We observe a significant difference in τ for high and low amplitude, which is explained by a duffing non-linearity in the equations of motion of the resonator. From ringdown measurements, we obtain a lower bound to the decay time of $\tau = 1.09 \cdot 10^5$ s. The error on the fit of this value is 14.7 seconds. At the moment, our understanding of this decay time of individual modes is that it depends strongly on the amplitudes in other modes due to a coupling

frequency [Hz]	tau [s]	Q factor
15.9	$3.65{\cdot}10^4$	$1.82 \cdot 10^6$
26.7	$1.09{\cdot}10^5$	$9.13 \cdot 10^6$
40.6	$1.43{\cdot}10^4$	$1.82 \cdot 10^6$
55.1	$3.37{\cdot}10^4$	$5.84 \cdot 10^6$
129	$0.214{\cdot}10^4$	$8.70 \cdot 10^5$
147	$0.152{\cdot}10^4$	$6.98 \cdot 10^5$

Table S1: Tabulated values of the resonator mode parameters. Again, these values are truncated at at three decimals, since the fit significance of these values is much higher than the actual stability of these values with respect to mode amplitude, as touched upon in the texts. The spring stiffness of the 26.7 Hz mode was calculated to be 12 mN/m.

of the non-linearities. This understanding is limited currently, and a further understanding would require further measurements. To minimise the effect of this false sense of precision (versus accuracy) in the current publication, we will instead truncate the values at three decimals. With the quality factor (Q) of the resonator defined as

$$Q = \pi f \tau \tag{S1}$$

and a frequency of the mode $f = 26.7\,\text{Hz}$, we obtain a quality factor of $Q = 9.06 \cdot 10^6$. From τ we can also determine the damping coefficient of the resonator, which is defined as

$$\gamma = \frac{2}{\tau} \tag{S2}$$

which gives a damping in the 27 Hz mode of $\gamma = 1.84 \cdot 10^{-5}\,\text{s}^{-1}$, or a linewidth of $\gamma/2\pi = 2.92\,\mu\text{Hz}$.

Supplementary Materials C Analysis and Calibration of the Single-Stage Particle Readout Circuit and Energy Coupling

The motion of the particle, and the resulting force noise of the particle modes, can be calibrated from flux to RMS motion by injecting a magnetic drive through the calibration transformer. The amount of magnetic flux injected is then directly quantised by the SQUID. By measuring the flux induced by the particle response and using the inductance in the circuit and the spring stiffnes, we find a calibration of magnetic flux to motion.

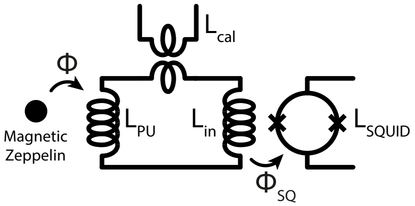

Fig. S5: **Schematic circuit of the single-stage readout circuit for the magnetic particle** . The transformer used to couple in the external flux used to calibrate the circuit is depicted at the top, with the particle and pick-up loop on the left, and the input coil and SQUID on the right.

This calibration can be intuitively derived from the energy coupling, β^2. The energy coupling is defined as the fraction of the energies in two coupled oscillating systems, i.e. the amount of energy that couples from one system to the other. This energy coupling is similar to the quality factor of a single system, which is defined as the fraction of energy lost per cycle with respect to the total energy in the system, or equivalently, a measure for the damping of the system. Conversely, the quality factor provides a measure for the maximal energy stored in the resonator as a fraction of the input energy when driven at resonance for a time $T \gg \tau$, that is to say: the energy at which the fractional energy loss per cycle is equal to the energy put in each cycle by the resonant drive.

For our system, with a mechanical resonator in the form of the trapped magnetic particle, and a driving magnetic field coupled in through the calibration transformer and coupled to the particle through the pick-up loop, we get

$$\beta^2 \equiv \frac{L_{total} I^2}{k x^2} \tag{S3}$$

with x any spatial coordinate. Going to the limit of infinitesimal displacement, and using $k = m\omega^2$ and using $L_{total} I^2 = \Phi^2 / L_{total}$, we obtain

$$\beta^2 = \frac{\left(\frac{d\Phi}{dx}\right)^2}{L_{total} \cdot m\omega^2} \tag{S4}$$

from which it is evident that the energy coupling can be used to determine the absolute motion of the magnetic particle from the flux measured in the SQUID.

To measure β^2, we further consider the motion of the resonator as a simple resonantly driven harmonic oscillator, with the *Ansatz* $x(t) = A(t)e^{i\omega t}$; $A(t) = \frac{F}{2m\omega} \cdot t$, from which we obtain a damping force

$$F_{damping} \left(= 2m\zeta\omega_0 \frac{dx}{dt} = \gamma_m v \right) = \gamma_m \cdot \omega \cdot x \tag{S5}$$

The quality factor Q is equivalently defined as $Q = \frac{1}{2\zeta}$, thus $\gamma_m = \frac{m\omega}{Q}$. In our calibration procedure, we inject a flux through the calibration transformer. This flux then results in a current through the detection circuit. This current is detected as a flux by the SQUID, with known $\frac{dI}{d\phi}$ calibration from the SQUID parameters. We refer to this current as the the crosstalk current $I_{crosstalk}$. This current also leads to a flux through the pick-up loop, which gives rise to a magnetic force on the particle of the form $F_{drive} = \alpha \cdot I_{crosstalk}$. Here, α is a coupling constant of units [N/A] that contains a geometrical factor determined by both the relative positioning of the particle with respect to the pick-up coil, and the physical sizes of this coil and particle. As the particle is driven, the motion of the particle will in turn induce a current in the detection circuit. This induced current has the form $I_{induced} = \delta \cdot x$ where δ is a coupling constant with units [A/m], which is by symmetry effected through the pick-up coil-particle geometry in the same way that α is.

Combining these results, and noting that for the steady state solution the driving force must be equal to the damping force

$$\frac{I_{induced}}{I_{crosstalk}} = \frac{\delta \cdot x}{F_{drive}/\alpha} = \frac{\delta \cdot \alpha \cdot x}{\gamma_m \cdot \omega \cdot x} = Q \cdot \frac{\alpha \cdot \delta}{m\omega^2} = Q \cdot \frac{\alpha \cdot \delta}{k} \tag{S6}$$

Since some of our decay times are rather long, in our experiments we work with a $Q_{eff} = \pi f T$, where T is the time during which the drive is applied. Whilst this is not the steady state, this discrepancy is fully absorbed in our definition of Q_{eff}:

$$\frac{I_{induced}}{I_{crosstalk}} = \frac{\delta \cdot x}{F_{drive}/\alpha} = \frac{\delta \cdot \alpha \cdot x}{\gamma_{eff} \cdot \omega \cdot x} = Q_{eff} \cdot \frac{\alpha \cdot \delta}{m\omega^2} = Q_{eff} \cdot \frac{\alpha \cdot \delta}{k} \tag{S7}$$

From this derivation, we have found that the fraction of the currents in our detection circuit contains all the coupling constants in our system. In fact, looking at the units, we find

$$\frac{\alpha \cdot \delta}{k} = \frac{\left[\frac{\mathrm{N}}{\mathrm{A}}\right] \cdot \left[\frac{\mathrm{A}}{\mathrm{m}}\right]}{\left[\frac{\mathrm{N}}{\mathrm{m}}\right]} \tag{S8}$$

Furthermore, for small displacements in x, the flux change through the pick-up loop can be treated as linear. The resultant current in the detection circuit must then be $I_{induced} = \frac{d\Phi}{dx} \cdot x \cdot \frac{1}{L_{total}}$ or $\delta = \frac{d\Phi}{dx} \cdot \frac{1}{L_{total}} \hat{=} \left[\frac{\mathrm{A}}{\mathrm{m}}\right]$.

As noted before, δ contains the same geometric scaling as α. From the symmetry, we recognise that $\alpha = \frac{d\Phi}{dx}$ ([Wb/m] = [N/A]). Combining these results, we find

$$\frac{\alpha \cdot \delta}{k} = \frac{\left(\frac{d\Phi}{dx}\right)^2}{L_{total} \cdot m\omega^2} = \beta^2 \tag{S9}$$

This final results gives us a measurable result for β^2, or, conversely, the proportionality constant $\frac{d\Phi}{dx}$ that equates our measured flux to an absolute motion. The calibration measure is then found from

$$\frac{d\Phi}{dx} = \sqrt{L_{total} \cdot m\omega^2 \cdot \beta^2} = \sqrt{L_{total} \cdot m\omega^2 \cdot \frac{I_{induced}}{Q \cdot I_{crosstalk}}} \tag{S10}$$

Noting that both $I_{induced}$ and $I_{crosstalk}$ are converted equally by the SQUID, the measured voltage can be inserted for the current through the detection circuit, which provides our sensitivity as

$$\frac{d\Phi}{dx} = \sqrt{L_{total} \cdot m\omega^2 \cdot \frac{\Delta V_{drive}}{Q \cdot V_{crosstalk}}} \tag{S11}$$

Here Φ is the flux through the pick-up loop. Since we detect this using the SQUID, we need to convert this value to the flux through the SQUID, which is done by dividing by the total inductance of the circuit and multiplying by the mutual inductance of the SQUID input loop and the SQUID loop. As a final step, this value can be related directly to the voltage measures made by taking the SQUID gain value as measured.

$$\frac{dV}{dx} = \frac{dV}{d\Phi_{SQ}} \cdot \frac{d\Phi_{SQ}}{dI} \cdot \frac{dI}{d\Phi} \cdot \frac{d\Phi}{dx} = \frac{dV}{d\Phi_{SQ}} \cdot M_{in,SQ} \cdot \frac{1}{L_{total}} \cdot \frac{d\Phi}{dx} \tag{S12}$$

The values for the inductances in $L_{total} = L_{PU} + L_{TP} + L_{input} + L_{calibration}$ are: $L_{PU} = 2.9 \cdot 10^{-7}$ H the inductance of the pick-up loop, $L_{TP} = 1 \cdot 10^{-7}$ H the inductance of the 10 cm superconducting twisted-pair connecting the SQUID input to the pick-up loop, $L_{input} = 4 \cdot 10^{-7}$ H the inductance of the input coil of the two-stage SQUID device, and $L_{calibration} = 2 \cdot 10^{-9}$ H the inductance of the small calibration transformer coil. For the mutual inductance, the SQUID

is calibrated to a value of $M_{in,SQ}^{-1} = 0.5\,\mu\mathrm{A}/\Phi_0$. The SQUID voltage gain is measured as $dV/d\Phi_0 = 0.43\,\mathrm{V}/\Phi_0$. These values, combined with measures of these ring-up's, gives us a value of $\frac{dV}{dx} = 0.16\,\frac{\mathrm{V}}{\mu\mathrm{m}}$ for the 27 Hz mode, with a relative error of 7%, which is dominated by the error in the voltage calibration measurement.

Supplementary Materials D Comparison to basic Optomechanical Quantities

Given the vast previous work in the field of optomechanics, we aim to provide a rough outline of how the work presented in this paper compares to quantities in optomechanical experiments, in this appendix. For a more complete treatise of optomechanics and specifically a comparison to magneto-mechanics, we refer to (29).

We note that the resonance frequency of our levitated particle corresponds to the frequency of the mechanical oscillator in an optomechanics experiment. At present, our experiment lacks the equivalent of the optical cavity and the light sent to the cavity. It could be added to the experiment, by changing the readout of our experiment. Currently, we use a SQUID based read-out, that relies on DC signals. We could read out the SQUID at higher frequencies (using a GHz cavity). In that case the inductance of the SQUID, which depends on the flux in the SQUID, would provide frequency tuning to a LC resonator, with typical resonance frequencies of 2-20 GHz.

We adopted ω to be the frequency of the mechanical resonator, this in contrast to optomechanics, in which Ω is nominally adopted for the mechanical frequency, with ω reserved for the optical frequency. In the rest of this appendix, we will refer to the optical frequency as used in optomechanics as ω_{opt} to avoid confusion. In the context of optomechanics, the optomechanical coupling, g, is given by the infinitesimal change in wavelength as a function of the change in position of the mechanical oscillator:

$$g_{opt} = \frac{d\omega_{opt}}{dx} \tag{S13}$$

This is an important quantity that provides a measure on how well the mechanical and optical systems are coupled. Often the value is quoted in terms of the zero-point-motion (x_{zpm}) of the resonator, as g_0. In the case of magnetic levitation, a translation from $\frac{d\Phi}{dx}$ to $\frac{d\omega_{opt}}{dx}$ can be made through the realization that the inductance of the SQUID $L_J = \frac{\Phi_0}{2\pi I_c(\Phi)}$ depends on the flux through the SQUID. This change in inductance changes the resonance frequency of the LC resonator, ω_{LC}, that could be used to readout our SQUID in the future. In the same way that the resonance frequency of an optical cavity depends on the position of a membrane, we also see that the resonance frequency of the LC circuit that the SQUID is part of, depends on the position of our levitated magnetic particle.

Combining this with equation S10, we find:

$$g_{mag} = \frac{d\omega_{LC}}{d\Phi}\frac{d\Phi}{dx} = \frac{d\omega_{LC}}{d\Phi}\sqrt{L_{total} \cdot m\omega^2 \cdot \beta^2} \tag{S14}$$

Evidently, this is the equivalent to g_{opt} for our SQUID detected system. Implicit in the term L_{total} is the coupling loss from parasitic inductances. Since the detection is not resonant, like it would be in an optical cavity system, this remains a negligible loss. Expressing g in terms of the resonator zero-point-motion can be done similar to how it is often derived in optomechanical systems, from equating the energy of the bosonic system at zero occupation to the kinetic energy at zero point motion:

$$x_{zpm} = \sqrt{\frac{\hbar}{2 \cdot m_{eff} \cdot \omega_m}} \tag{S15}$$

Where m_{eff} is the effective mass of the mechanical oscillator and ω_m is the frequency of the mechanical oscillator. In our case the m_{eff} is just the mass of the levitated particle and ω_m is the eigenfrequency of the measurement. Filling in the mass and frequency used in the gravity measurements, a zero point motion of $x_{zpm} = 0.86\,\mathrm{fm}$ can be calculated. Combining this with our measured sensitivity, we find $\frac{d\Phi}{dx} \cdot x_{zpm} = 63\,\mathrm{n}\Phi_0$ as a zero point flux for our system. For a slope, $\frac{d\omega_{LC}}{d\Phi}$, of a $\lambda/4$ resonator terminated with a SQUID of $1\,\mathrm{GHz}/\Phi_0$, this results in a $g_{0,mag} = g_{mag} \cdot x_{zpm} = 63\,\mathrm{Hz}$. We conclude, however, that it will be a considerable challenge to make the Q factor of such a LC resonator high enough to reach the sideband resolved limit.

Supplementary Materials E Correction and Conversion of the Data to Units of N/$\sqrt{\text{Hz}}$

The data used in our gravitational experiments comes from the photodiode detector which measures the passages of the masses on the wheel, and the SQUID signal. Both these signals are filtered by our lock-in amplifiers. Since our gravity experiments were performed at differing amplitudes in the mode, each time trace had a different slope, as discussed in Supp. B. To account for this, we have subtracted an individual exponential decay in the form of $Ae^{t/\tau_{fit}}$ from each.

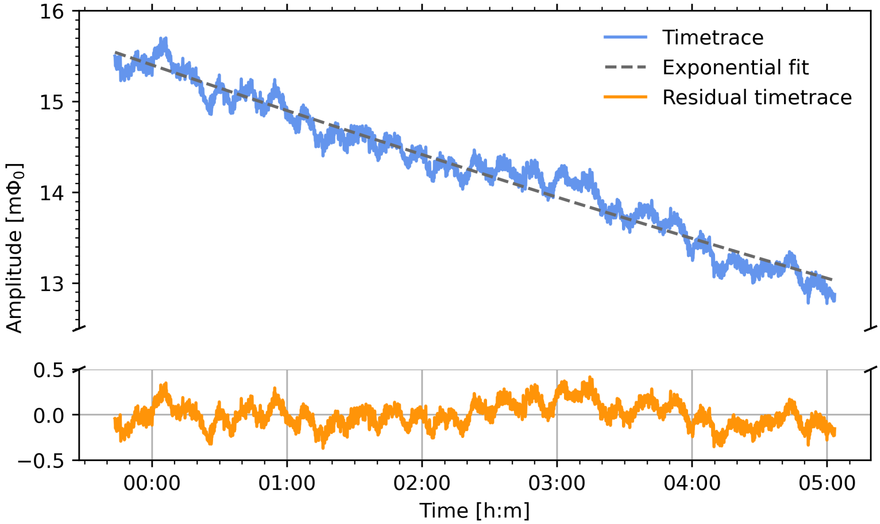

Fig. S6: **Ringdown subtraction as performed on the time trace measurement of each wheel position**. Shown here for a separation between particle and driving mass at wheel phase zero of 48.1 cm vertical, and laterally displaced by 3.5 cm. Residual plotted in the lower half, where we see the fluctuations as result of the drive up and drive down effected by the mass-wheel, which is slightly detuned in frequency with respect to the mode. We drive detuned since we wish to stay away from the non-linear driving and frequency shift of the mode that happens at large amplitude. Furthermore, detuned driving enables us to substract the absolute amplitude.

After subtracting the ringdown, we apply a phase factor to the time traces. This phase factor is determined based on the detuning of the resonator frequency with respect to the lock-in center frequency. This change ensures that

the central peak of the resonator mode will fall fully in a single bin of the Fourier transform we perform next, this ensures that the transfer function of the resonator mode is fully symmetrical. Before performing the Fourier transform, we also cut out a section of the time trace in which there is an integer amount of phase cycles for the mass-passage signal, which ensures smooth periodic boundary conditions for our FFT. This results in a spectrum giving us the motion of the particle per root hertz, which can be converted to RMS motion in a specific frequency band by integrating over that frequency bandwidth.

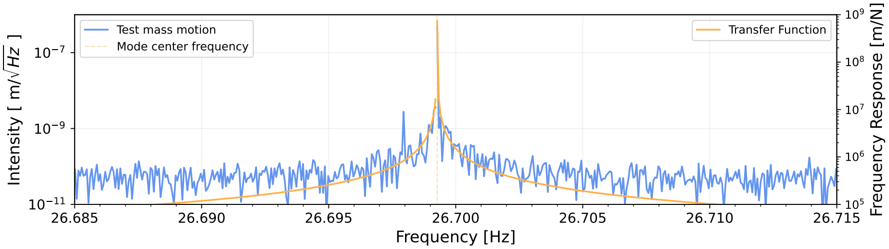

Fig. S7: **Spectrum of the time trace converted to motional noise, with the transfer function plotted overlaid**. The orange vertical line indicates the resonance frequency of the mode.

By then subtracting the transfer-function of the mechanical mode from the particle signal, and applying our conversion factor to go from Φ_0 to displacement, and using a spring stiffness $k = m\omega^2$ and a mode bandwidth of $df = Q/f$, we arrive at our force noise spectrum in $\mathrm{N}/\sqrt{\mathrm{Hz}}$

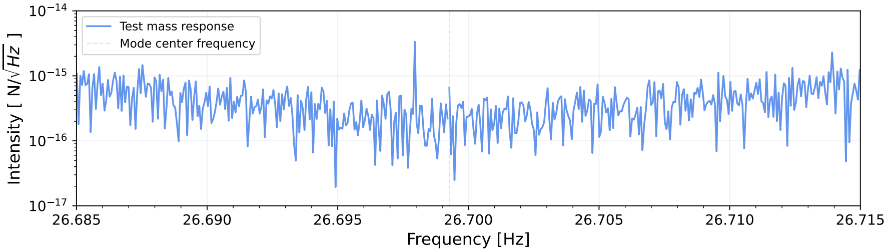

Fig. S8: **Force noise spectrum**. Spectrum of the time trace converted to force noise, the final product of this procedure. The orange vertical line indicates the resonance frequency of the mode.

The noise floor of this measurement corresponds to a mode temperature of 3 K, from $T_{mode} = kx^2_{RMS}/k_B$.
